# Sustainment of a complex culturally competent care intervention for Hispanic living donor kidney transplantation: A longitudinal analysis of adaptations

**DOI:** 10.1017/cts.2022.378

**Published:** 2022-03-28

**Authors:** Elisa J. Gordon, Jefferson J. Uriarte, Naomi Anderson, Justin Dean Smith, Juan Carlos Caicedo, Michelle Shumate

**Affiliations:** 1 Northwestern University Feinberg School of Medicine, Chicago, IL 60611, USA; 2 Spencer Fox Eccles School of Medicine at the University of Utah, Salt Lake City, UT, 84132, USA; 3 Northwestern University, Evanston, IL 60208, USA

**Keywords:** CFIR, Equity, Fidelity, FRAME, Health disparities, Implementation science, Interview

## Abstract

**Introduction:**

Sustainment refers to continued intervention delivery over time, while continuing to produce intended outcomes, often with ongoing adaptations, which are purposeful changes to the design or delivery of an intervention to improve its fit or effectiveness. The Hispanic Kidney Transplant Program (HKTP), a complex, culturally competent intervention, was implemented in two transplant programs to reduce disparities in Hispanic/Latinx living donor kidney transplant rates. This study longitudinally examined the influence of adaptations on HKTP sustainment.

**Methods:**

Qualitative interviews, learning collaborative calls, and telephone meetings with physicians, administrators, and staff (n = 55) were conducted over three years of implementation to identify HKTP adaptations. The Framework for Reporting Adaptations and Modifications-Expanded was used to classify adaptation types and frequency, which were compared across sites over time.

**Results:**

Across sites, more adaptations were made in the first year (n = 47), then fell and plateaued in the two remaining years (n = 35). Adaptations at Site-A were consistent across years (2017: n = 18, 2018: n = 17, 2019: n = 14), while Site-B made considerably fewer adaptations after the first year (2017: n = 29, 2018: n = 18, 2019: n = 21). Both sites proportionally made mostly skipping (32%), adding (20%), tweaking (20%), and substituting (16%) adaptation types. Skipping- and substituting-type adaptations were made due to institutional structural characteristics and lack of available resources, respectively. However, Site-A’s greater proportion of skipping-type adaptations was attributed to greater system complexity, and Site-B’s greater proportion of adding-type adaptation was attributed to the egalitarian team-based culture.

**Conclusion:**

Our findings can help prepare implementers to expect certain context-specific adaptations and preemptively avoid those that hinder sustainment.

## Introduction

Organizations are commonly unable to maintain implementation of evidence-based interventions (EBIs) over time [1–3]. Sustainment – the continued delivery of implementation strategies over time while continuing to produce intended outcomes despite planned or unplanned intervention adaptations [4] – is an important facet of translational science aimed at preventing the decline and termination of EBIs [5,6]. Adaptations refer to purposeful changes to the design or delivery of an intervention to improve its fit and effectiveness within a specific context [7]. Because little is known about factors contributing to sustainment, interventionists may not be adequately prepared to encounter adaptations [8,9], particularly the dynamic nature of ongoing adaptations [10].

Examining sustainment can help predict and increase the likelihood of long-term EBI delivery [8,10,11]. Factors contributing to sustainment include less intervention complexity (e.g., smaller number of interacting components) [12], ample funding and available resources (e.g., staff, time), and ability to adapt intervention components [3,13–15]. The few studies examining reasons for intervention adaptations reported enhancing engagement in the intervention, reaching specific audiences, and increasing intervention fit to the institution [16,17]. Studies examining sustainment of complex interventions found that poor fit within an existing organizational infrastructure led to less likelihood of sustainment [12,18]. Research on the sustainment of hospital-based surgical care programs 3 to 6 years after successful implementation found that adaptations made to better meet patients’ needs were associated with program sustainment [14].

While there are a variety of study designs for sustainment research, scholars recommend assessment over multiple time points throughout a multi-year study to identify potential changes to the intervention that may affect sustainment [3,5,19]. Such an approach contributes to understanding the dynamic nature of complex intervention sustainment over the long term [7,10,20]. However, few studies have done this [21]. Moreover, despite calls for examining the sustainment of culturally competent transplant interventions [22,23], to our knowledge few studies have done so [24].

The Northwestern Medicine™ Hispanic Kidney Transplant Program (HKTP) was established in December 2006 to provide culturally competent and linguistically congruent care to Hispanic/Latinx patients initiating evaluation for kidney transplantation, with the intent of increasing living kidney donation rates [25]. Living donor kidney transplantation offers greater patient and transplant survival compared to deceased donor kidney transplantation [26]. However, Hispanics/Latinx patients are less likely to obtain living donor kidney transplants compared to non-Hispanic White patients due to healthcare system, provider, and patient-level factors [27]. HKTP addresses these multi-level factors contributing to disparities. HKTP was associated with a 47% increase in living donor kidney transplantation among Hispanic patients at one of two intervention sites to no detriment to other ethnic/racial groups [28].

HKTP was implemented at two transplant programs and evaluated for effectiveness and implementation fidelity. While most complex interventions occur for one or two years [29], our intervention spanned three years. In this paper, we examine the effect on HKTP sustainment during this period by identifying the frequency, type, and instigator of adaptations and differences between implementing sites.

### Theoretical Frameworks

The Framework for Reporting Adaptations and Modifications-Expanded (FRAME) [7] guided the evaluation of intervention adaptations. The FRAME is a coding system designed to classify the types of adaptations made to interventions to understand how they influence intervention implementation. The FRAME incorporates eight features: (1) when in the implementation process the adaptation occurred, (2) whether the adaptation was planned or unplanned, (3) by whom are adaptations made, (4) what is modified, (5) at what level of delivery the adaptation is made, (6) type of context or content-level adaptations, (7) the extent to which the adaptation is fidelity-consistent, and (8) the reasons for the adaptation, including the goal or intent of the adaptation and contextual factors that influenced the decision. The Consolidated Framework for Implementation Research (CFIR) [30] guided the implementation design and data collection tools. CFIR includes 39 constructs in 5 domains: intervention characteristics, organizational inner setting, characteristics of individuals, outer setting, and process [30,31].

## Materials and Methods

### Study Design

We conducted a type II hybrid effectiveness-implementation study design [32] to assess HKTP at two sites. The pre-implementation period occurred from January 1, 2011, to December 31, 2016, while the intervention period occurred from January 1, 2017, to March 15, 2020, and ended early due to the COVID-19 pandemic. This paper examines intervention adaptations and their influence on HKTP sustainment during the three-year implementation phase. We compared adaptations made in the first year (2017) to those made in 2018 and 2019. Because the study ended prematurely due to COVID-19, we did not include data from the first quarter of 2020 to focus on comparisons by year and by site. We used the Template for Intervention Description and Replication [33] and the Consolidated Criteria for Reporting Qualitative Research for quality reporting [34]. Northwestern University’s Institutional Review Board approved the study (STU00201331).

### Setting and Participants

HKTP was implemented at two kidney transplant programs: Site-A, located in the southern region of the USA, and Site-B, located in the southwest region. Both sites were selected because they served large Hispanic populations, perform more than 50 LDKTs per year, and have a Hispanic bilingual transplant physician. Two matched control sites were used for comparison.

Eligible participants were stakeholders directly involved in HKTP implementation and included transplant physicians (surgeons, nephrologists, urologist), administrators (administrative directors, managers), and clinical and research staff (nurses, social workers, schedulers, and marketing). Site PIs notified and requested stakeholders to participate in data collection activities via email, phone, and/or in-person. Stakeholders did not have a relationship with the co-PIs (EJG, JCC) prior to study commencement. Co-PIs presented reasons for performing the research with site PIs prior to grant submission and to all stakeholders at the initial site visit.

### Intervention and Effectiveness

As a multi-level intervention, HKTP is comprised of multiple components and strategies, as shown elsewhere [28]. HKTP provides the same standard of care to patients receiving transplant evaluation while using a different care delivery process [35]. System-level HKTP intervention components include a bicultural/bilingual social worker performing outreach to and rapport building with Hispanic patients at dialysis centers and subsequent coordination with scheduling staff to set up patient appointments. Provider-level components include delivering in-person transplant education to patients by bilingual and bicultural transplant physicians who are regarded as authoritative figures by many Hispanic people and involving bilingual/bicultural spoken and written communication throughout. Patient-level components include addressing cultural beliefs and concerns pertaining to organ donation and transplantation through cultural salient idioms during education sessions in a group format that fosters greater comfort with learning.

We used several implementation strategies including learning collaboratives, in-service presentations within each site, marketing events, and data monitoring. The research team held quarterly “Learning Collaboratives” involving stakeholders from both sites to design solutions (including intervention adaptations) to barriers to implementing HKTP [36]. Organizations and providers use this quality improvement approach to accelerate learning by sharing experiences and best practices [37]. Additionally, biweekly telephone calls with research coordinators at each site were conducted to review study progress and intervention adaptations. Sites were encouraged to engage in in-service presentations within each site to raise providers’ awareness of HKTP and increase referrals. Marketing events outside the institution were conducted to similarly raise awareness among external providers as well as patients and families. Sites were also guided to engage in data monitoring of HKTP’s implementation. However, the research team did not systematically track the use of data monitoring strategies but inquired into its occurrence during interviews. Thus, it is unclear to what extent sites engaged in monitoring.

We also monitored sites’ progress in establishing HKTP during the pre-implementation period by providing a monthly timeline that compared progress made at each site. Using green, yellow, and red colored indicators of site progress on each implementation step, the timeline was intended to be informative and foster motivation through competition. This approach was discontinued several months after sites began implementing because it was designed to foster program launch and not sustainment.

In the primary outcomes paper for this study [28], we found that HKTP effectively increased LDKT in Hispanic patients at one of two intervention sites, in comparison to pre-/post-assessments at two matched control sites. The study included 2,063 recipients. Site-A improved the Hispanic LDKT rate by 47% (from 20.3% at pre-HKTP to 29.8% at post-HKTP), Site-A exhibited greater fidelity to the protocol than Site-B, which may partially explain why Site-A observed intervention effects during the study; additional findings are described elsewhere [28]. Further, our previous research (manuscript under review) [38] found that CFIR inner setting factors (i.e., structural characteristics, culture, compatibility, available resources) influenced the initial adaptations of HKTP. In addition, the transplant team made most of the adaptations. The current research extends these findings by examining changes to HKTP over time and their effect on sustainment.

### Fidelity Measures

To assess HKTP implementation fidelity, research staff documented the a) number of in-service and marketing materials and events used to promote HKTP, b) number of dialysis centers visited and number of hours spent performing outreach, c) bilingual and bicultural status of outreach staff, d) audio-recorded quarterly phone calls with patients scheduling their transplant evaluation visit, e) audio-recorded quarterly HKTP education sessions to document the core content areas covered, f) number of education sessions held and the type of clinician delivering the sessions, g) number of potential recipients and family members attending education sessions, h) whether each potential recipient received a call from a physician 6–8 weeks after the education session to complete transplant evaluation, and i) number of patients who were sent 90-day reminder letters to complete transplant evaluation.

### Data Collection

Qualitative data were collected during annual site visits in 2017, 2018, and 2019. Study co-PIs (EJG, an experienced female social scientist, JCC, a male transplant surgeon) conducted the visits, which involved one-on-one interviews and group discussions with transplant stakeholders to identify barriers and facilitators to HKTP implementation, assess intervention delivery, and troubleshoot ways to facilitate HKTP implementation.

Co-PI EJG conducted in-depth, semi-structured interviews with stakeholders to assess perceptions of organizational culture, attitudes about the implementation complexity, and perceived barriers and facilitators to implementing HKTP components using the CFIR Interview Guide (www.cfirguide.org). Interview questions included the following: “What kinds of changes have you had to make to your outreach plan to fit with your institutional setting/city/demographics?”, “What kind of changes do you think that you will (still) need to make to the implementation so it will work more effectively in your setting?”, “What kind of data is your organization collecting on the HKTP in how well it’s doing or working?”, and “How hard is it going to be to sustain the HKTP?”. The co-PIs led in-person group discussions to clarify the study protocol, assess progress on intervention implementation, and brainstorm ways to accommodate the intervention.

Written informed consent was obtained for interviews, while verbal informed consent was obtained for group discussions and learning collaborative meetings, all of which were audio-recorded and lasted 20–190 min; the wide range was due to one learning collaborative workshop occurring as a 1½ day in-person retreat. Written field notes were taken during all data collection activities.

### Qualitative Analysis

Audio recordings were transcribed and coded using a two-step process. First, two of four research staff members independently coded each transcript for HKTP intervention components, adaptations, and stakeholder perceptions of sustainment. After achieving inter-rater reliability on a subset of transcripts (Kappa > 0.70), all transcripts were recoded. Discrepancies between coders were resolved through group discussions. Second, two research staff members independently coded the same transcript for adaptations using the FRAME, identifying the type, instigator, and reason for the adaptation [7]. Coding discrepancies were solved through group discussion. Recoded transcripts were uploaded into qualitative analysis software (MAXQDA v.12).

### Quantitative Analysis

Descriptive statistics were used to calculate frequencies of adaptations by study site, demographics, adaptation type, instigator, and reason. Analyses were conducted with the Statistical Packages for Social Sciences (SPSS) version 26.

## Results

### Demographics

Fifty-five stakeholders (Site-A: n = 29, Site-B: n = 26; 100% recruitment rate) participated in one or more of the following research activities across all three years: site visit interview (n = 48), group discussion (n = 18), and/or learning collaborative discussion (n = 22) (Table 1). Most participants were female (80%), non-Hispanic (55%), and included non-physician clinicians (e.g., nurses, social workers) (22%), administrators (18%), physicians (9%), and other staff (e.g., schedulers, research staff, marketing) (51%).

**Table 1.** Participants’ demographic characteristics by study site, 2017 – 2019

Characteristic	Total N (%) N = 55	Site-A N (%) N = 29^*^	Site-B N (%) N = 26^**^
Gender			
Female	44 (80)	25 (86)	19 (73)
Male	11 (20)	4 (14)	7 (27)
Ethnicity			
Non-Hispanic	30 (55)	14 (48)	16 (62)
Hispanic	25 (45)	15 (52)	10 (38)
Training			
Non-Physician Clinician	12 (22)	5 (17)	7 (27)
Staff: Scheduler	12 (22)	7 (24)	5 (19)
Administrator	10 (18)	6 (21)	4 (15)
Staff: Research	8 (15)	5 (17)	3 (12)
Staff: Marketing	7 (13)	2 (7)	5 (19)
Physician	5 (9)	3 (10)	2 (8)
Staff: Front Desk	1 (2)	1 (3)	0

* Site-A had 13 participants in 2017, 21 in 2018, and 20 in 2019. The total represents the number of unique participants.

** Site-B had 16 participants in 2017, 17 in 2018, and 18 in 2019. The total represents the number of unique participants.

### Intervention Components

#### HKTP Attendance

A comparable number of potential recipients attended HKTP sessions at Site-A (n = 159) and Site-B (n = 152) (Table 2). Site-A reported an increase of potential recipients over time (2017 = 43, 2018 = 55, 2019 = 61), while Site-B reported a decrease in 2018 followed by an increase in 2019 (2017 = 50, 2018 = 46, 2019 = 56). Site-B reporter a greater number of family members attending HKTP education sessions than Site-A (n = 196; Site-B: n = 267). However, Site-B reported a decrease in family member attendance over time (2017 = 119, 2018 = 79, 2019 = 69), while Site-A reported an increase after the first year of implementation (2017 = 57, 2018 = 73, 2019 = 66).

**Table 2.** Adherence to the HKTP protocol over time

	Total	2017	2018	2019
	N (%)	N (%)	N (%)	N (%)
Number of Marketing Materials^1^				
Site-A	14	8	4	2
Site-B	15	6	7	2
Number and Proportion of Marketing Materials Translated Into Spanish^1,^^*^				
Site-A	9 (64)	5 (63)	3 (75)	1 (50)
Site-B	7 (47)	1 (17)	5 (71)	1 (50)
Number of In-Service Presentations^1^				
Site-A	6	3	1	2
Site-B	3	1	0	2
Number of Marketing Events^1^ for Providers				
Site-A	23	11	6	6
Site-B	1	1	0	0
Number of Marketing Events^1^ for Patients				
Site-A	36	14	12	10
Site-B	3	2	1	0
Number of Hours Performing Outreach to Dialysis Centers and Proportion of Hours that Met Target Goals^2,^ ^3^				
Site-A	1,455 (58)	390 (47)	529 (64)	536 (64)
Site-B	689 (28)	278 (33)	164 (20)	247 (30)
Number of Dialysis Centers Visited^1^				
Site-A	705	245	292	168
Site-B	147	49	45	53
Bilingual Outreach Staff^4^				
Site-A	1 (66)^5^	0	1 (100)	1 (100)
Site-B	1 (100)^5^	1 (100)	1 (100)	1 (100)
Bicultural Outreach Staff^4^				
Site-A	1 (66)^5^	0	1 (100)	1 (100)
Site-B	0	0	0	0
Non-Bilingual, non-Bicultural Outreach Staff^4^				
Site-A	1 (100)^5^	1 (100)	1 (100)	1 (100)
Site-B	1 (66)^5^	1 (100)	1 (100)	0
Number and Adherence to Scheduler/Nurse Telephone Scheduling Script^6^				
Site-A [range]^7^	40 (93) [67–100]	12 (89) [69–100]	20 (95) [67–100]	8 (96) [88–100]
Site-B [range]^7^	12 (88) [60–100]	5 (84) [63–100]	4 (85) [60–100]	3 (97) [90–100]
Number of Education Sessions Held and Proportion of Sessions that Met Target Goals^1,^ ^8^				
Site-A	66 (92)	21 (88)	23 (96)	22 (92)
Site-B	57 (79)	21 (88)	21 (88)^9^	15 (63)^9^
Number and Proportion of Education Sessions Delivered by Physician^1^				
Site-A	66 (100)	21 (100)	23 (100)	22 (100)
Site-B	47 (82)	19 (90)	16 (76)^10^	12 (80)
Number of Potential Recipients Attending Education Sessions^1^				
Site-A	159	43	55	61
Site-B	152	50	46	56
Number of Family Members Attending Education Sessions^1^				
Site-A	196	57	73	66
Site-B	267	119	79	69
Number of Family Members Attending Education Sessions Per Patient				
Site-A	1.25	1.33	1.33	1.08
Site-B	1.78	2.38	1.72	1.23
Number and Proportion of Patients Sent 90-Day Reminder Letters to Complete Transplant Evaluation^11^				
Site-A	34 (21)	7 (16)	17 (31)	10 (16)
Site-B	2 (1)	2 (4)	0	0
Number and Proportion of Patients Who Received a Call from a Physician 6–8 weeks After the Education Session to Complete Transplant Evaluation^11^,^12^,^13^				
Site-A	21 (18)^14^	9 (23)	6 (15)	6 (15)
Site-B	34 (25)^15^	14 (31)	10 (23)	10 (20)

* Denominator was the number of marketing materials within each year.

1 Data were obtained from the REDCap Project: Metrics.

2 Data were obtained from the REDCap Project: Budget Impact Analysis.

3 Sites aimed to conduct 832 h of outreach per year.

4 The HKTP protocol required at least one staff member to fit this characteristic.

5 Number of unique outreach staff who fit this characteristic from 2017 to 2019.

6 Only a subset of scheduling calls was audio-recorded; Site-A recorded 3 calls per month, while Site-B recorded one per quarter.

7 Range refers to the lowest and highest percentages by which schedulers and physicians adhered to the education session presentation points or telephone scheduling script.

8 Sites aimed to hold 24 HKTP education sessions per year.

9 Site-B canceled 1 HKTP clinic in 2018, and 6 HKTP clinics in 2019, because Site-B’s IRB had lapsed.

10 Four education sessions were initially delivered by a nurse then completed by a physician.

11 Data were obtained from the REDCap Project: Hispanic Kidney Transplant Program.

12 Site-A had 41 patients who completed the transplant evaluation before the 6–8 week physician phone call.

13 Site-B had 15 patients who completed the transplant evaluation before the 6–8 week physician phone call.

#### Transplant Patient Education Sessions

Sites aimed to offer 24 HKTP clinics per year for a total of 72 HKTP clinics. Overall, Site-A delivered a greater number and proportion of education sessions (n = 66, 92%) than Site-B (n = 57, 79%). Site-A closely adhered to the protocol each year of the intervention period (2017 = 88%, 2018 = 96%, 2019 = 92%). Site-B adhered to the protocol the first two years of the HKTP intervention, but adherence decreased in 2019 (2017 = 88%, 2018 = 88%, 2019 = 63%).

The HKTP protocol requested that all education sessions be delivered by a bilingual, bicultural physician. Site-A reported a greater number and proportion of HKTP education sessions delivered by a bilingual, bicultural physician (n = 66, 100%) than Site-B (n = 47, 82%). Over time, all HKTP education sessions were delivered by a bilingual, bicultural physician at Site-A (2017 = 100%, 2018 = 100%, 2019 = 100%), while Site-B had a lower proportion (2017 = 90%, 2018 = 76%, 2019 = 80%).

#### Implementation Strategies Needed for Sustainment

For HKTP to be sustainable, sites must continue to recruit new potential recipients through outreach efforts at dialysis centers, schedule potential HKTP recipients, and conduct culturally competent education sessions. They must also maintain a bicultural/bilingual team and the availability of interpreters or interpreter services. The implementation strategies used in this study also suggest that ongoing training, in-service presentations to local transplant stakeholders, marketing campaigns, ongoing data monitoring for quality assurance, and learning collaboratives might be necessary, but the pre-post non-randomized study design did not allow formal testing [28].

#### Learning Collaborative Meetings

During the pre-implementation period, one 4-hour learning collaborative was held by telephone, and one 1½ day in-person retreat, which entailed discussion to: clarify the intervention protocol, brainstorm approaches to align the intervention with institutional context, train providers through role-playing sessions, and review progress made. During the implementation period, twelve 1-hour telephone meetings were held from 2017–2019 (range: 3–5 per year).

#### In-Service Presentations

Across all years, Site-A hosted more in-service presentations than Site-B (n = 6 versus n = 3). Both sites reported a drop in in-service presentations in 2018 and an increase in 2019 (Site-A: 2017 n = 3, 2018 n = 1, 2019 n = 2; Site-B: 2017 n = 1, 2018 n = 0, 2019 n = 2).

#### Marketing

Across all years, Site-A hosted more marketing events targeting healthcare providers at other institutions than Site-B (n = 23 versus n = 1). Both sites hosted the most events in 2017, which dropped and remained constant in subsequent years (Site-A: 2017 n = 11, 2018 n = 6, 2019 n = 6; Site-B: 2017 n = 1, 2018 n = 0, 2019 n = 0).

Across all years, Site-A hosted more marketing events targeting patients and family members than Site-B (n = 36 versus n = 3). Both sites hosted most events in 2017, which dropped in subsequent years (Site-A: 2017 n = 14, 2018 n = 12, 2019 n = 10; Site-B: 2017 n = 2, 2018 n = 1, 2019 n = 0).

#### Outreach

Sites aimed to complete 832 h of outreach at dialysis centers per year for a total of 2,496 h during the study period, as per the protocol. Overall, Site-A dedicated more hours and completed a greater proportion of the target goal (n = 1455, 58%) than Site-B (n = 689, 28%) (Table 2). Site-A increased the time spent on outreach after the first year (2017 = 390, 2018 = 529, 2019 = 536), while Site-B decreased the time spent in the second year but increased its outreach efforts in the third year (2017 = 278, 2018 = 164, 2019 = 247). Site-A’s increase over the years coincided with an increase in potential recipients attending HKTP. By contrast, Site-B’s decreased outreach efforts in 2018 resulted in an 8% decrease in HKTP attendance; once Site-B increased its outreach efforts in 2019, Site-B reported a 22% increase. Although Site-A spent more time conducting outreach than Site-B, both sites reported a similar number of potential recipients attending HKTP.

The HKTP protocol called for intervention sites to have a bilingual, bicultural staff member lead outreach efforts at dialysis centers to effectively communicate and recruit potential HKTP recipients. Site-A did not hire a bilingual/bicultural outreach staff member until 2018, while Site-B’s outreach efforts were led by a bilingual staff who was not bicultural. While a non-bilingual, non-bicultural staff member led Site-A’s outreach efforts in 2017, Site-A reported fewer potential recipients attending HKTP than Site-B, despite dedicating more outreach time than Site-B. When Site-A hired a bilingual, bicultural staff member in 2018, they experienced a 28% increase in HKTP attendance. Because Site-B’s bilingual outreach staff performed outreach all three years, the decrease in HKTP attendance in 2018 may not have been caused by the outreach staff’s bilingual and bicultural status, but rather time spent performing outreach.

#### Scheduling

To assess adherence to the scheduler scripts, sites recorded a subset of calls. Site-A recorded 3 calls per month, while Site-B recorded one call per quarter. Overall, both sites reported comparable adherence (Site-A: n = 40, 93%; Site-B: n = 12, 88%). Over time, both sites increased adherence after the first year (Site-A: 2017 = 89%, 2018 = 95%, 2019 = 96%; Site-B: 2017 = 84%, 2018 = 85%, 2019 = 97%). At Site-A, the increase coincided with an increased number of potential recipients attending HKTP education sessions. By contrast, despite the increase in adherence over time, Site-B experienced an 8% decrease in attendance in 2018, which may be a result of its decreased outreach efforts. In 2019, Site-B reported its highest adherence to scheduler scripts which coincided with its highest number of potential HKTP recipients. Overall, sites’ adherence to the scheduler scripts resulted in comparable HKTP attendance numbers.

#### Data Monitoring

Although stakeholders (site PIs) were encouraged to monitor their site data at the study onset, they indicated during site visit interviews that they did not collect or monitor the study-specific data. Stakeholders reported that they lacked awareness of processes for tracking participants involved in the study at Site-A and that tracking participants would have required additional time to manually prepare the list of known participants at Site-B.

### Adaptations

**Fig. 1.**
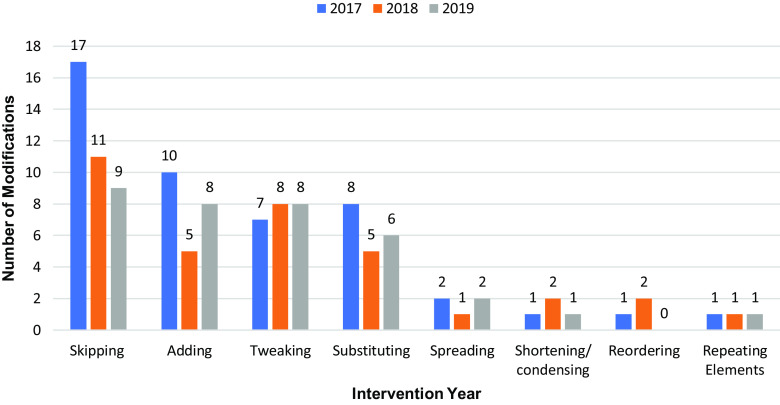
Adaptation types over time: sites combined.

Across sites, more adaptations were made in the first year (2017: n = 47) and then declined and plateaued over the two remaining years (2018: n = 35, 2019: n = 35) (Table 3). Overall, the most frequent adaptation types included the following: 1) skipping/delaying, which refers to the removal or delay of intervention components, 2) adding, which refers to the addition of new components (e.g., materials, activities) to the intervention, 3) tweaking, which are minor changes to intervention components, and 4) substituting, which refers to the replacement of intervention components with other components not consistent with the original intervention. While the frequency of skipping/delaying, adding, and substituting-type adaptations decreased over time, tweaking-type adaptations remained relatively consistent over time (Fig. 1).

**Table 3.** Frequency of adaptations by initiator, type, and study site

	Grand Total	Year 1 - 2017			Year 2 - 2018			Year 3 - 2019		
	N = 117 N (%)	Total N	Site-A N (%)	Site-B N (%)	Total N	Site-A N (%)	Site-B N (%)	Total N	Site-A N (%)	Site-B N (%)
Number of Adaptations	117	47	18 (38%)	29 (62%)	35	17 (49%)	18 (51%)	35	14 (40%)	21 (60%)
Adaptation Initiator										
Transplant Team	75 (64)	30 (64)	12 (66)	18 (62)	24 (69)	12 (71)	12 (60)	21 (60)	8 (57)	13 (62)
Individual	24 (21)	9 (19)	3 (17)	6 (21)	5 (14)	4 (23)	1 (6)	10 (29)	5 (36)	5 (24)
Institution	15 (13)	6 (13)	3 (17)	3 (10)	6 (17)	1 (6)	5 (28)	3 (9)	0	3 (14)
More than one	3 (3)	2 (4)	0	2 (7)^1^	0	0	0	1 (3)	1 (7)^2^	0
Adaptation Types										
Skipping	37 (32)	17 (36)	9 (50)	8 (28)	11 (31)	5 (29)	6 (33)	9 (26)	4 (29)	5 (24)
Adding	23 (20)	10 (21)	2 (11)	8 (28)	5 (14)	3 (18)	2 (11)	8 (23)	5 (36)	3 (14)
Tweaking	23 (20)	7 (15)	3 (17)	4 (14)	8 (23)	6 (35)	2 (11)	8 (23)	3 (21)	5 (24)
Substituting	19 (16)	8 (17)	3 (17)	5 (17)	5 (14)	2 (12)	3 (17)	6 (17)	0	6 (29)
Spreading	5 (4)	2 (4)	1 (6)	1 (3)	1 (3)	0	1 (6)	2 (6)	1 (7)	1 (5)
Shortening	4 (3)	1 (2)	0	1 (3)	2 (6)	0	2 (11)	1 (3)	1 (7)	0
Reordering	3 (2)	1 (2)	0	1 (3)	2 (6)	1 (6)	1 (6)	0	0	0
Repeating Elements	3 (2)	1 (2)	0	1 (3)	1 (3)	0	1 (6)	1 (3)	0	1 (5)
Lengthening	0	0	0	0	0	0	0	0	0	0
Loosening Structure	0	0	0	0	0	0	0	0	0	0
Changes in materials	0	0	0	0	0	0	0	0	0	0
Integrating another treatment	0	0	0	0	0	0	0	0	0	0
Drift	0	0	0	0	0	0	0	0	0	0

1 Two adaptations were initiated by both the institution and an individual in 2017.

2 One adaptation was initiated by the institution and the transplant team in 2019.

**Fig. 2.**
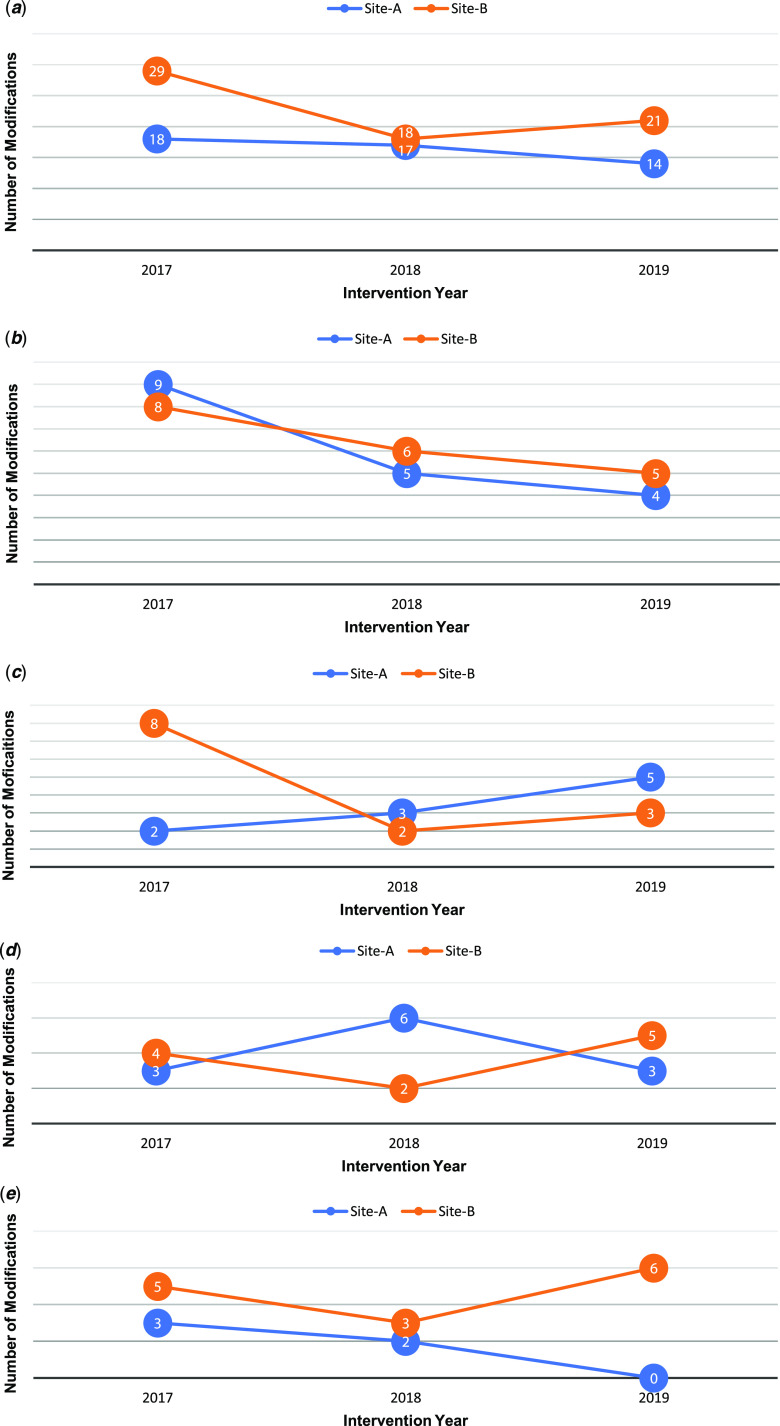
a. Total adaptations by study site and intervention year. b. Skipping/delaying adaptations by study site and implementation year. c. Adding adaptations by study site and implementation year. d. Tweaking adaptations by study site and implementation year. e. Substituting adaptations by study site and implementation year.

Site-B consistently made more adaptations than Site-A over time (Table 3, Fig. 2a). While Site-A made approximately the same number of adaptations across all three years, Site-B made fewer adaptations from 2017 to 2018 but made slightly more in 2019.

#### Skipping/delaying

Both sites made a comparable number of skipping/delaying-type adaptations (Site-A: n = 18; Site-B: n = 19) (Fig. 2b). Both sites decreased the number of delaying/skipping-type adaptations from 2017 to 2018 and were consistent from 2018 to 2019. In 2017, sites experienced delays in preparing Spanish HKTP materials, did not implement a Spanish-speaking transplant telephone line, and did not implement a HKTP quality assurance tracking system, as prescribed in the protocol. Site-A also did not hire a bilingual, bicultural staff member to lead outreach at dialysis centers. In 2018, study sites engaged in fewer delays in preparing Spanish HKTP materials. Further, schedulers at Site-A did not offer the HKTP intervention to all potential patients, while Site-B underwent a change in its hospital-wide electronic health record system, which halted outreach efforts for 3 months and delayed patient scheduling. In 2019, institutions continued to experience delays in patient scheduling and Site-B did not conduct wrap-up sessions. In 2019, there was no mention of delays pertaining to developing HKTP materials.

#### Adding

Overall, Site-A made fewer adding-type adaptations than Site-B (Site-A: n = 10 Site-B: n = 13) (Fig. 2c). Adding-type adaptations were designed to improve outreach efforts, education sessions, and assist patients. In 2017, both sites provided laptops to Spanish-speaking potential living donors to assist in completing the English BREEZE Transplant^TM^ (an online medical history form). At Site-A, stakeholders added information to education slides and included town hall meetings to introduce HKTP to potential patients and increase outreach efforts. Site-B included Spanish-speaking transplant professionals (e.g., nurses, transplant surgeon) at education sessions to answer questions from Spanish-speaking patients. Further, Site-B created flyers to inform potential HKTP patients at dialysis centers of upcoming outreach sessions and provided telephone assistance to potential living donors when completing the English Breeze. In 2018, Site-A’s previous additions to improve outreach efforts and education sessions continued. By contrast, in 2018, Site-B discontinued its effort to enhance outreach by announcing upcoming dialysis clinic visits through flyers and stopped assisting Spanish-speaking potential living donors to complete the Breeze but continued to have transplant professionals in attendance at education sessions. In 2019, Site-A continued and increased its efforts to improve outreach by adding a “mini-automatic enrollment” procedure to expedite the scheduling of potential HKTP patients.

Site-B made more adding-type adaptations than Site-A, likely because it made more efforts to improve patient engagement (e.g., transplant professionals at education sessions) and to assist Spanish speakers in the first year. However, Site-B discontinued 2017 additions to improve outreach (e.g., outreach flyers) and to assist Spanish-speaking potential living donors, which resulted in a decrease of adding-type adaptations in 2018. By contrast, Site-A continued 2017 additions made to improve outreach efforts, which increased over time. Both sites made adding-type adaptations to address patient needs. Site-A made additions to its outreach efforts to provide accessible information to its Spanish-Speaking patients, while Site-B made additions to increase patient engagement during education sessions and to provide patient support.

#### Tweaking

Over time, both sites made a comparable number of tweaking-type adaptations (Site-A: n = 11; Site-B: n = 12) (Fig. 2d). Site-A made more from 2017 to 2018 and made fewer from 2018 to 2019. By contrast, Site-B made fewer from 2017 to 2018 and made more adaptations from 2018 to 2019. Overall, both sites made tweaking-type adaptations to improve patient scheduling and education sessions. Each year, both sites tweaked the schedulers’ script to improve its delivery and feasibility for staff. Sites also tweaked education slides and adjusted the time of education sessions to improve delivery, patient engagement, and patient family attendance. While both sites made tweaking-type adaptations to improve patient scheduling and education sessions, Site-B decreased the time spent on outreach in 2017 and 2018 because the outreach staff had other competing demands and scheduled fewer patients than prescribed by the protocol because the site lacked the space to accommodate more patients and their family members in 2017.

#### Substituting

Site-A made fewer substituting-type adaptations than Site-B (Site-A: n = 5; Site-B: n = 14) (Fig. 2e). Site-A made slightly fewer over time. Site-B made slightly fewer from 2017 to 2018 but made twice as many in 2019 than in 2018. Over time, both sites relied on Spanish-speaking staff to serve as interpreters.

While a bilingual/bicultural outreach staff was hired in 2018 at Site-A, the English-speaking outreach staff continued to do outreach. While the English-speaking outreach staff was involved in outreach at dialysis facilities (directed toward facility staff) in all 3 years, the Spanish-speaking outreach staff led patient-directed outreach efforts. Further, the English-speaking staff was accompanied by a Spanish-speaking research staff to help interpret. To improve communication with Spanish-speaking patients, Site-A relied on bilingual research and outreach staff to act as interpreters because there were not enough available interpreters from 2017 to 2018. While Site-B also relied on Spanish-speaking staff members to serve as interpreters, Site-B also relied on Spanish-speaking staff to conduct education and wrap-up sessions because there were not enough available Spanish-speaking physicians throughout the three years of implementation. Site-B made more substituting-type adaptations in 2019 than previous years because there were less Spanish-speaking staff available to assist with scheduling and clinical staff follow-ups and thus relied on a Spanish-speaking research staff.

Both sites made substituting-type adaptations because of lack of available resources (e.g., Spanish-speaking staff). Site-B made more substituting-type adaptations than Site-A because it had fewer available Spanish-speaking staff. While Site-B made a relatively consistent number in 2017 and 2018, it increased the number of substitutions made in 2019 because it needed more Spanish-speaking staff to conduct patient scheduling and clinical staff follow-ups which resulted in a Spanish-speaking research staff fulfilling those roles.

## Discussion

While the conceptualization of sustainment continues to evolve, most frameworks and models include multiple dimensions thought to be related to, or necessary for, ongoing intervention delivery over multiple years [2,10,15]. One commonality is intervention adaptation. Initial adaptation is likely undertaken to ensure fit of the intervention with a specific implementation context [30], whereas ongoing adaptation reflects attempts to align the intervention with an ever-changing delivery system and policy context [2,10]. This study focused on intervention adaptations but can also shed some light on the implementation strategies that also need to continue to ensure fidelity-consistent adaptations required both for sustained delivery and the maintenance of intervention effectiveness.

Both study sites made a number of adaptations of different types to HKTP over time. Adaptations of the adding type tended to increase over time in attempts to increase the effectiveness of the intervention. Substituting and delay/skipping-type adaptations decreased over time suggesting a potential need earlier in implementation to prune or de-implement HKTP components that were ineffective or misaligned with the context of the delivery sites. Relatively speaking, Site-B made fewer adaptations early in the implementation, which might have led to poorer fidelity and the need for more adaptations in the third year to increase effectiveness. This degree of adaptations might be expected given that HKTP was developed at Northwestern Medicine^TM^, and this study represented the first attempt to scale up the intervention to other systems.

Notable intervention adaptation differences emerged between the sites. Site-A, which had higher implementation fidelity than Site-B, made fewer adaptations across all years of implementation. Further, tweaking or substituting types of adaptations were more likely to increase over time, as occurred at Site-B. While adaptation is expected when new interventions are implemented in a particular setting [10], this finding suggests that HKTP did not align well with the context of Site-B given both the amount and type of adaptations (i.e., more adding), coupled with lower fidelity that assuredly contributed to the lack of effectiveness at this site. Intuitively, one might conclude that the adaptations were fidelity-inconsistent per the FRAME [7], or perhaps that certain barriers in the setting were simply intractable, and despite ongoing efforts to change HKTP to fit the setting, changes to the context were also needed to accommodate the intervention – a conceptualization aligning with the Dynamic Sustainability Framework [10].

The use of the learning collaborative strategy was hypothesized to help ensure adaptations were both fidelity-consistent and would increase the implementation and effectiveness of HKTP through cross-site learning between Sites A and B and Northwestern University, where the HKTP was developed and first implemented. While this seems to have worked well for Site-A, it was not sufficient to overcome contextual barriers in Site-B. Evidence of the effectiveness of learning collaboratives for implementation has been mixed, in large part due to the high degree of protocol variability [39–41]. This study only included two sites and 12 learning collaborative meetings, which limited our ability to understand precisely why site differences emerged as a result of the learning collaborative. A deeper dive into the site differences could provide support for continued use of the learning collaborative for long-term sustainment, but it is unclear from this study the extent to which this strategy was instrumental to sustainment in comparison to other strategies and factors. One consideration is the degree to which the study team had the authority and influence to alter the context. Adapting the intervention was the primary purview of the study team, but findings suggest that involving additional stakeholders with the power to change contextual factors, primarily in the inner setting, might have been needed.

Despite encouragement to use data monitor strategies, both sites indicated not doing so. Similarly, a review of healthcare organizations implementation of culturally competent interventions found that relatively few studies implemented audit or quality improvement approaches of the intervention [42]. Had sites monitored study data throughout the implementation period, stakeholders would have likely noticed subtle ways that adaptations affected intervention effectiveness. Further, one could anticipate that the stakeholders involved in monitoring would become more engaged in the study and that the intervention would have become more embedded into the institution’s inner setting. All these aforementioned factors may have fostered sustainment.

Although sustainment has become recognized as an essential implementation outcome [6], there may be challenges to its evaluation. While it was helpful to monitor fidelity, sustaining this practice in the long term is unlikely given limited stakeholder bandwidth to carry out this additional work. Ultimately, the decision about monitoring fidelity depends on the priority that intervention sites devoted to the intervention. This also suggests a need to create more feasible and sustainable fidelity monitoring methods [43].

A strength of this study is a longitudinal and ongoing analysis of adaptations to a complex, culturally competent intervention using the FRAME over a three-year period across diverse healthcare organizations. Limitations include reliance on participant self-reports, which may not reflect actual behaviors. Social desirability bias may have affected descriptions of adaptations. However, data triangulation through stakeholder interviews and weekly phone calls with research staff served to clarify and confirm adaptations. Future research could conduct observational evaluations of adaptations, but this is often infeasible due to cost and the nature of the intervention. The two-site design limits our ability to draw conclusions regarding the effects of specific strategies on intervention adaptations and sustainment more broadly. Future research should examine the extent to which adaptations beget other adaptations. Doing so would likely necessitate a more frequent evaluation that could capture the dynamics of adaptation, as well as the use of other implementation strategies, using tools such as the Longitudinal Implementation Strategy Tracking System [44,45]. Last, contemporary models of implementation sustainment are multidimensional and include factors such as funding, leadership support, organizational capacity, and workflow integration, among others [5,15]. Our primary focus on adaptations, while rigorous, is but one contributor to sustainment.

Our findings illuminate factors that challenge the sustainment of complex interventions and underscore the importance of ongoing assessment of adaptations concerning their impact on target outcomes. Further, certain contextual determinants challenge the sustainment of complex interventions that cannot be overcome solely through adapting the intervention. Chasing fit with context by adapting the intervention led to low-fidelity implementation in one site, and certain adaptations had a clear adverse effect on intervention delivery. For example, lack of time and available Spanish-speaking staff led to adaptations, which resulted in outreach not being performed at target levels.

Systematic reviews show that culturally competent care interventions are effective in improving patient, provider, access, and organizational outcomes [42,46,47]. Few culturally competent care interventions have evaluated their sustainment [48]. We anticipate that maintaining the HKTP’s bilingual/bicultural staffing requirement may present a challenge to the HKTP’s sustainment. Both transplant programs noted that it was difficult to find bilingual/bicultural staff.

Practically, our findings can help prepare implementers to expect certain context-dependent adaptations to preemptively avoid those that reduce fidelity and effectiveness and to focus on those that support sustainment. The manner through which such decisions for adaptations are made remains a question for future research but our findings suggest the potential for learning collaboratives to play a role in these processes.
